# Insights and priorities from the 2025 private funders’ Parkinson's disease and related disorders large footprint data interoperability summit

**DOI:** 10.1177/1877718X261430266

**Published:** 2026-03-10

**Authors:** Leslie C Kirsch, Emily Baxi, Mukta Phatak, Francis Jeanson, Dave Alonso, Cornelis Blauwendraat, Matthew CH Boersma, Patrick Brannelly, Matthew HS Clement, Amy Easton, Hilary Jenkins, Ritu Kapur, Tyler Mollenkopf, Bryce Pickard, Amy Rommel, Carol L Thompson, Jonathan White, Sonya B Dumanis

**Affiliations:** 1 The Michael J. Fox Foundation for Parkinson's Research, New York, NY, USA; 2 The Milken Institute, Washington, DC, USA; 3 The 10,000 Brains Project, Oakland, CA, USA; 4 Centre for Analytics, The Ontario Brain Institute, Toronto, ON, USA; 5 Coalition for Aligning Science, Chevy Chase, MD, USA; 6 Aligning Science Across Parkinson's (ASAP), Chevy Chase, MD, USA; 7 FBRI, San Francisco, CA, USA; 8 The Alzheimer's Disease Data Initiative, Kirkland, WA, USA; 9 Target ALS, New York, NY, USA; 10 Hiro Studio, Calgary, Alberta, Canada; 11 Bridge Analytics, South San Francisco, CA, USA; 12 The Allen Institute for Brain Science, Seattle, WA, USA; 13 The Ontario Brain Institute, Toronto, ON, USA; 14 Rainwater Charitable Foundation, Fort Worth, TX, USA

**Keywords:** FAIR data, data interoperability, open data, big data, findability

## Abstract

Effective sharing of large datasets across traditionally siloed research domains has the power to transform conventional research practice dramatically, but has been stymied by persistent barriers to data access and interoperability. The 2025 Private Funders’ Parkinson's Disease and Related Disorders (PDRD) Data Interoperability Summit brought together technical leaders from North American-based private funders and research organizations engaged in large-scale neurodegenerative data efforts to address these challenges through the lens of FAIR (Findability, Accessibility, Interoperability, and Reusability) principles. Pre-summit activities included structured interviews, comprehensive data assessments, and preparatory exercises to identify common pain points, systemic gaps, and areas of opportunity across the data ecosystem. During the summit, participants collaboratively developed and prioritized a suite of actionable recommendations, underscoring the urgency and complexity of improving the neurodegenerative data ecosystem. Despite facing significant technical, legal, regulatory, and cultural barriers - ranging from high data management costs and privacy concerns to fragmented governance structures - participants expressed strong alignment on the need for strategic, equitable, and collaborative solutions. Here, we summarize the emerging recommendations from those discussions as well as the high-priority initiatives selected for immediate action across the funding agencies. These efforts mark a critical first step in addressing longstanding barriers and reflect a shared commitment to advancing collaborative data sharing. Continued work in this area promises to accelerate discovery and innovation, with the potential to drive significant breakthroughs in the understanding, diagnosis, and treatment of neurodegenerative diseases.

## Plain language summary

This review explores non-profit funder approaches to reducing technical, policy, and cultural barriers to data interoperability across Parkinson’s disease and related disorders to improve research data findability, accessibility, interoperability, and reliability. Consensus prioritized recommendations from the group are presented, along with proposed action plans for interested stakeholders.

## Introduction

Neurodegenerative diseases, including Parkinson's disease (PD), Alzheimer's disease (AD), and Amyotrophic Lateral Sclerosis (ALS), along with related conditions such as Frontotemporal Dementia (FTD), Huntington's disease, Lewy Body Dementia, Progressive Supranuclear Palsy (PSP), Corticobasal Degeneration (CBD), and Chronic Traumatic Encephalopathy (CTE), pose significant global health challenges, affecting millions worldwide and imposing substantial social and economic burdens. PD exists along a spectrum of these neurodegenerative disorders, sharing overlapping features with related conditions. These shared characteristics reflect common underlying biological mechanisms and highlight the complexity of disease presentation and progression.

Progress in understanding the underlying causes, developing early and accurate diagnostic tools, and finding effective treatments has increasingly depended on the ability of researchers to access, integrate, and analyze large-scale datasets from diverse cohorts and studies. Further, an increasing body of literature points to the importance of engaging in co-pathology investigation, given that often multiple diseases appear on a spectrum and there is high clinical and pathological overlap.^1–7^ Therefore, it is key to understand the complex relationship between neurodegenerative diseases. The availability of large-scale datasets to facilitate cross-cutting analysis across multiple diseases is of particular importance in improving trial design^8^ and advancing precision medicine approaches to treatment.^9^ Yet, despite considerable investments in data collection, data management, and technological advancement, the research community continues to face significant hurdles in achieving effective data interoperability both within disease-specific datasets and across the broader spectrum of neurodegenerative conditions.^10^

Sharing data through public repositories is associated with higher citations of primary papers, suggesting benefits for researchers and data contributors who choose to deposit data for sharing with the larger community.^11–13^ As the value of data sharing gains broader recognition, challenges related to data interoperability are increasingly coming to the forefront—underscoring the need to ensure that poor-quality or inconsistent data doesn’t compromise the utility and impact of downstream integrated analyses. Data interoperability is defined as the capacity for diverse data systems and organizations to seamlessly exchange, integrate, and utilize information in a standardized manner, and it is crucial for maximizing the scientific value of existing datasets and accelerating the pace of discovery. In 2016, the FAIR principles (Findability, Accessibility, Interoperability, and Reusability) were introduced to address the growing need for structured and efficient data sharing in scientific research.^14^ See Table 1 for a high-level overview of the FAIR principles. These principles have emerged as the benchmark framework guiding data-sharing practices, promoting more efficient use of resources, reducing redundancy, and enabling collaborative research efforts. However, the practical implementation of these principles remains challenging, largely due to technical complexities, high costs, cultural resistance, and challenges related to compliance with legal requirements and privacy regulations.

**Table 1.** FAIR data sharing principles.^14^

Principle	Formal definition	Layman's terms
Findability	Data and metadata are assigned globally unique and persistent identifiers and are described with rich metadata to support discovery.	Ensuring that data can be reliably discovered by both humans and machines
Accessibility	Metadata and data are retrievable by their identifier using a standardized communication protocol, with authentication and authorization where necessary.	Providing clear, reliable access to data and associated documentation, in accordance with legal, ethical, and security considerations
Interoperability	Data uses a formal, accessible, shared, and broadly applicable language for knowledge representation.	Facilitating the integration and exchange of information across diverse platforms through the use of shared standards and formats.
Reusability	Data are richly described with accurate and relevant attributes and released with a clear and accessible data usage license to support replication and reuse.	Supporting data reuse by ensuring clarity, completeness, and consistency in both the data and its associated metadata

Research data management specialists across various disciplines and organizations have long lamented the difficulty in effectively providing FAIR-compliant services to the research community. A 2024 Milken Institute report highlighted the gap in governmental funding for infrastructure necessary to de-silo individual neurodegenerative diseases’ datasets in service of artificial intelligence for precision medicine.^15^ A more recent 2025 report of challenges faced by data repository managers recommended that research funders can help address this gap by (1) investing in shared infrastructure for cross-institutional data management, (2) convening disciplinary communities to build consensus, and (3) developing mechanisms to support long-term cloud storage costs.^16^ Recognizing the urgent need to address these issues, in particular for the large footprint data on PD and related disorders already emerging, The Michael J. Fox Foundation (MJFF), in partnership with Aligning Science Across Parkinson's (ASAP) and the Coalition for Aligning Science (CAS), convened and organized the 2025 Private Funders’ Parkinson's Disease and Related Disorders Data Interoperability Summit for private funders based in North America who are sponsoring and funding the collection and management of multi-study large footprint datasets (e.g., multi-’omics, raw imaging, digital health technologies). The purpose of the summit was to identify meaningful opportunities for collaboration to align and improve research data sharing among a small group of organizations with overlapping large-scale data collection and management efforts. Eleven organizations were represented in this effort. Representatives from these organizations were a mix of technical and policy professionals, ranging from data scientists and engineers to legal and compliance experts to program and project managers. See Table 2 for a list of the organizations represented.

**Table 2.** Overview of the organizations represented at the 2025 PDRD data interoperability summit.

Organization	Who we are
Allen Institute	We advance fundamental neuroscience through open science, team-based research and data sharing.
Alzheimer's Disease Data Initiative (ADDI)	We are dedicated to accelerating research and discovery in Alzheimer's disease and related dementias by fostering data sharing and collaboration. We offer researchers around the world secure data sharing, analytics, and collaboration tools and resources, all available to users at no cost.
Aligning Science Across Parkinson's (ASAP)	A research initiative focused on addressing resource and knowledge gaps in the development and progression of Parkinson's disease, with a goal of amplifying and coordinating efforts around the world within and outside the existing PD community. This initiative is managed by the Coalition for Aligning Science and Implemented through the Michael J Fox Foundation.
Coalition for Aligning Science (CAS)	An organization dedicated to designing and implementing large-scale biomedical research programs to accelerate discoveries. The Aligning Science Across Parkinson's (ASAP) is managed under CAS.
Bridge Analytics	We believe in a future in which a deep biological understanding of Parkinson's disease, bipolar disorder, and profound autism enables patients to receive personalized care that significantly lessens the burden of these diseases. We are building the analytic infrastructure and collaborative ecosystem needed to recognize this vision.
FBRI	We believe that therapeutic breakthroughs are achieved through discoveries in disease biology, the development of transformative technologies, and increased information exchange.
Milken Institute	Through the Science Philanthropy Accelerator for Research and Collaboration (SPARC), we work to develop, launch, and lead initiatives that propel scientific and medical research.
Michael J Fox Foundation (MJFF)	We are dedicated to speeding the development of better treatment and ultimately finding a cure for Parkinson's Disease.
Ontario Brain Institute (OBI)	We drive innovation and collaboration in neuroscience research to improve brain health and quality of life for individuals with neurological conditions. Public and controlled multimodal data from numerous brain health domains are available via OBI's Brain-CODE platform.
Rainwater Charitable Foundation	We support groundbreaking research to develop treatments and cures for neurodegenerative diseases, with a focus on tauopathies such as FTD, PSP, CBD, and CTE.
Target ALS	We are dedicated to breaking down barriers in ALS research by providing funds and core resources to researchers in industry and academia worldwide.
The 10,000 Brains Project	We aim to advance understanding of how neurodegenerative diseases develop and progress by leveraging advanced tools—such as AI—and connecting insights across conditions. Our goal is to drive earlier detection, more precise diagnosis, and better outcomes for patients through data-driven, cross-disease research.

Collectively, these organizations actively invest in over 35 different data collection efforts supporting data repositories to digitally archive and curate data collections, data portals to promote searchability within a collection, and knowledge platforms to provide integrated tools, visualizations, ontologies, and computational resources to assist users with interpreting and synthesizing the data. See Table 3 for an overview of the different curated knowledge and data access portals supporting FAIR-Aligned Neurodegenerative Disease Research that these organizations are actively involved with.

**Table 3.** Overview of the Various curated knowledge and data access portals supporting FAIR-aligned neurodegenerative disease research that the organizations collectively support through funding or active data management.

Curated knowledge and data access portals	Tool type	Description of data collection
*Data Repositories: Digital storage systems designed to archive, curate and preserve data sets*		
Zenodo ASAP Data Repository^17^	Data Repository	open-access platform that hosts and shares Parkinson's disease research datasets contributed by the ASAP Initiative to promote transparency and data reuse.
Zenodo MJFF Data Repository^18^	Data Repository	open-access platform that hosts and shares Parkinson's disease research datasets contributed by MJFF researchers to promote transparency and data reuse.
*Data Portals: Primary function is an interface to access and search harmonized or single-study data*		
Allen Brain Observatory^19^	Data Portal	standardized in vivo survey of physiological activity in the mouse visual cortex.
Alzheimer's Disease Workbench^20^	Data Portal	centralized platform that integrates diverse datasets, analytical tools, and visualization resources to accelerate data sharing and collaborative research in Alzheimer's disease and related dementias.
Accelerating Medicines Partnership Parkinson's Disease and Related Disorders (AMP-PDRD)^21^	Data Portal	public and private sector partners to generate, integrate, and share large-scale clinical, imaging, and molecular data to accelerate biomarker discovery and therapeutic development for Parkinson's disease and related disorders.
BioFIND^22^	Data Portal	cross-sectional, observational study that provides high-quality clinical and biomarker data from moderately advanced Parkinson's disease patients and healthy controls to support biomarker discovery and validation.
Center for Alzheimer's Dementia (CARD)^23^	Data Portal	open-access, multi-omic, imaging, and clinical datasets from deeply phenotyped cohorts to support research into the mechanisms, diagnosis, and treatment of Alzheimer's disease and related dementias.
CRN Cloud^24^	Data Portal	secure, cloud-based data platform that enables researchers to access, analyze, and share Parkinson's disease ‘omic data sets collaboratively while ensuring data privacy and regulatory compliance.
Dementias Platform UK (DPUK)^25^	Data Portal	national research platform that integrates and provides access to diverse longitudinal cohort data to accelerate dementia research and facilitate collaboration across the scientific community.
Fox Insight^26^	Data Portal	large-scale, online observational study collecting self-reported clinical and lifestyle data from people with and without Parkinson's disease to advance understanding of disease progression and heterogeneity.
Global Neurodegeneration Proteomics Consortium (GNPC)^27^	Data Portal	a public–private partnership compiling one of the largest neurodegeneration-focused proteomics datasets—∼250 million protein measurements from ∼35,000 biosamples across multiple diseases with harmonized clinical data.
Global Parkinson's Genetics Program (GP2)^28^	Data Portal	an international consortium aiming to expand understanding of Parkinson's disease genetics by collecting and analyzing diverse genomic and clinical data from populations worldwide.
LRRK2 Cohort Consortium^29^	Data Portal	global collaborative effort that gathers clinical, biomarker, and genetic data from individuals with LRRK2 gene variants to improve understanding of Parkinson's disease mechanisms and guide therapeutic development.
LRRK2 Investigative Therapeutics Exchange^30^	Data Portal	collaborative network that facilitates sharing of data, resources, and expertise to accelerate the development of targeted therapies for Parkinson's disease linked to LRRK2 genetic mutations.
Ontario Neurodegenerative Disease Research Initiative (ONDRI)^31^	Data Portal	multi-site longitudinal study that collects comprehensive clinical, imaging, genetic, and biomarker data to investigate common and distinct features across neurodegenerative diseases.
Parkinson's Progression Markers Initiative^32^	Data Portal	large, multicenter observational study collecting longitudinal clinical, imaging, and biospecimen data to identify biomarkers that track Parkinson's disease progression and support therapeutic development.
Path-ND^33^	Data Portal	next-generation platform designed to digitize, centralize and democratize access to the largest compilation of whole image slides of human brain tissue for research into neurodegenerative diseases.
Target ALS Data Engine^34^	Data Portal	natural history, multi-omic, and postmortem data sets derived from ALS patients to support research into disease mechanisms and biomarker discovery.
Tau Consortium^35^	Data Portal	curates multi-modal datasets—including genomic, proteomic, imaging, and clinical data—focused on understanding tau-related neurodegenerative diseases to accelerate biomarker and therapeutic discovery.
UK Biobank^36^	Data Portal	large-scale biomedical resource containing extensive genetic, clinical, imaging, and lifestyle data from half a million UK participants to support research into a wide range of diseases, including neurodegeneration.
*Knowledge Platform, a platform designed to to synthesize and interpret data by offering integrated tools, visualizations, ontologies and computational resources*		
Allen Brain Cell Atlas^37^	Knowledge Platform	multimodal single-cell reference atlas enabling exploration of over 20 million brain cells (mouse and human), combining transcriptomic, electrophysiological, and morphological data.
Allen Brain Map^38^	Knowledge Platform	integrated portal offering datasets, tools, and resources enabling researchers to investigate brain function, gene expression, and connectivity, including the Seattle Alzheimer's Disease Brain Cell Atlas.
Allen Brain Reference Atlases^39^	Knowledge Platform	high-resolution, annotated maps of brain anatomy across species—including mouse, human, and non-human primates—providing standardized frameworks for spatially mapping gene expression, connectivity, and cell types.
Brain Knowledge Platform^40^	Knowledge Platform	integrative resource developed to organize, visualize, and explore brain data across multiple modalities, including the Cell Type Knowledge Explorer
Critical Path for Parkinson's^41^	Knowledge Platform	Integrated clinical, biomarker, and genetic datasets designed to accelerate drug development and regulatory approval processes for Parkinson's disease therapies.
EBRAINS Knowledge Graph^42^	Knowledge Platform	neuroscience data platform that integrates diverse datasets, models, and tools to support data-driven brain research and facilitate collaboration across disciplines.
European Platform for Neurodegenerative Diseases (EPND)^43^	Knowledge Platform	connects large-scale datasets and biobanks across Europe to accelerate research and biomarker discovery in neurodegenerative diseases.

Over two days, participants identified key challenges and developed practical recommendations for advancing FAIR-aligned data-sharing practices, aiming to build strategic momentum for broad and sustained changes.

## Methodology for recommendation development and prioritization

**Figure 1.**
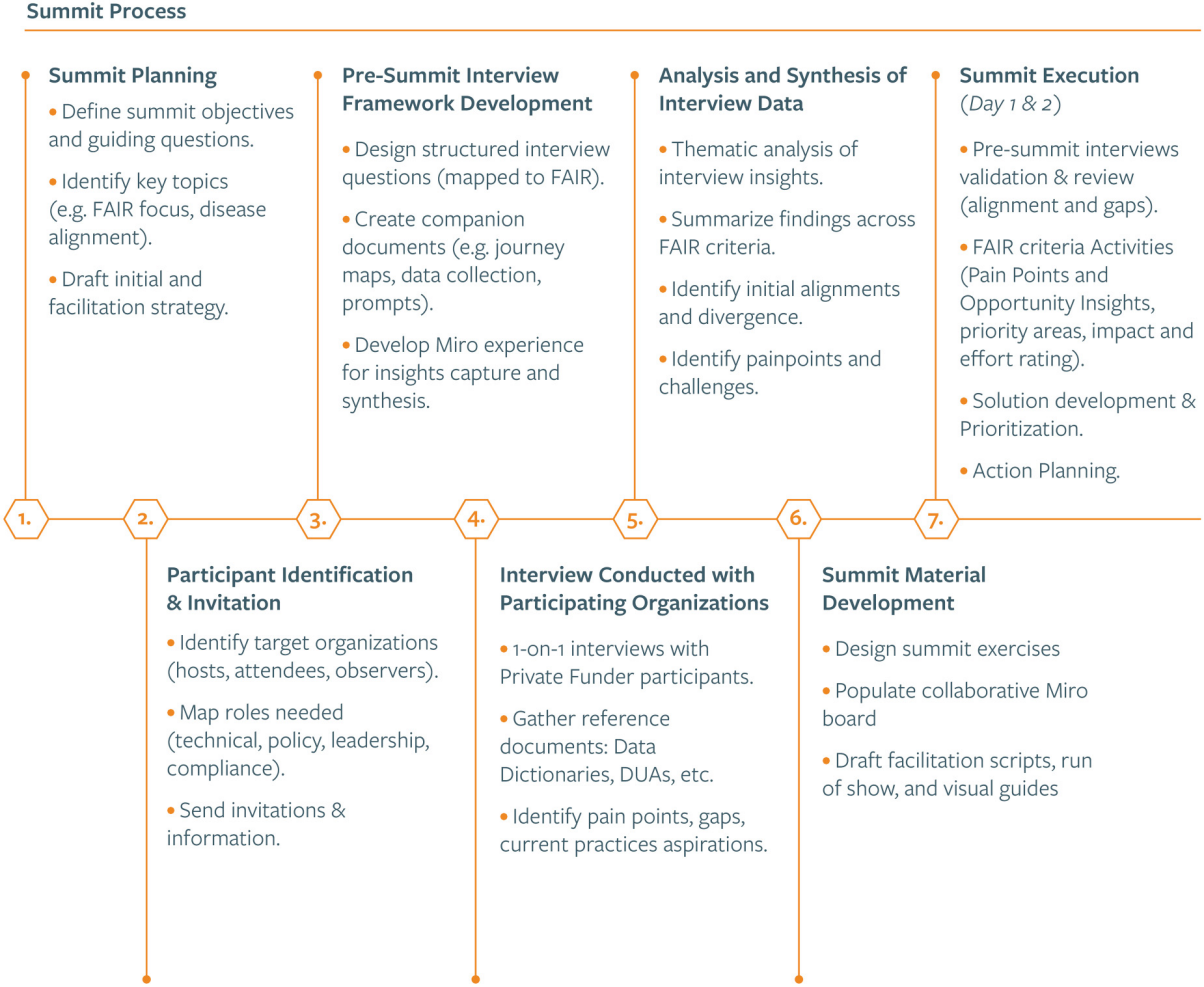
Summary summit preparation and execution processes.

To maximize productivity and relevance, extensive pre-summit activities were conducted to identify technical, policy, and cultural barriers, pain points, and opportunities for improvement across each FAIR principle. Workshop facilitators conducted interviews with each participating organization to explore specific challenges, existing practices, and potential solutions related to their own experiences in supporting large data collection and management efforts; see Supplemental Figure 1 for an example semi-structured interview template. Participants also provided crucial documentation—including Data Dictionaries, Data Use Agreements (DUAs), and existing governance frameworks—which informed a comprehensive analysis and guided summit preparations. See **Figure 1** for an overview of our process.

Insights from these interviews and documents were synthesized into targeted materials, visual frameworks, and journey maps that served as foundational tools during the summit. The two-day event, held in New York City, employed highly interactive methodologies—including facilitated discussions, structured breakout sessions, and collaborative exercises—to drive engagement and surface actionable recommendations. Collaborative digital tools such as Miro^44^, enabled real-time visualization and documentation of participant input, enhancing shared understanding and iteration. Attendees were encouraged before the summit to reflect on potential technical, policy, and cultural barriers to FAIR-aligned data sharing. See Table 4 for how we defined the three barrier categories.

**Table 4.** Technical, policy, and cultural components of FAIR data sharing within PDRD research.

Category	Definition
Technical	Infrastructure and resource constraints, which impact the ability of data contributors and users to fully realize the intended benefits of open data sharing.^45^
Policy	Expectations and guidelines set by stakeholders throughout the FAIR data sharing ecosystem, which mandate or influence the behaviors of researchers engaged in data sharing activities.^46^
Cultural	Human-centric influencing factors which may facilitate or constrain research data management activities, e.g., acceptance, demand, incentives, or communications.^47^

During the summit, participants validated and refined pre-summit findings, ensuring an accurate and comprehensive view of current challenges for each FAIR element - Findability, Accessibility, Interoperability, and Reusability. Recommendations to address the challenges raised for each FAIR element were developed and refined through structured breakout discussions, multivoting, and scoring exercises. Recommendations were then prioritized using a structured 2 × 2 matrix assessing impact and implementation effort. See Supplemental Table 1 for a complete list of recommendations, including each recommendation's estimated impact and implementation effort. In the final phase, participants developed detailed action plans for top-voted priority initiatives, outlining action plans to emphasize co-ownership, with participants committing not just to ideation but to implementation leadership. The summit concluded with shared reflections, a commitment to ongoing coordination, and a roadmap for reporting progress.

## Identified opportunities and challenges

The summit revealed a landscape of both complexity and convergence across the FAIR data-sharing criteria. The sections below present detailed findings by each FAIR principle, and where funders identified potential opportunities.

### Findability: ensuring that data can be reliably discovered by both humans and machines

**Shared Challenges:** Data repositories are commonly siloed, and metadata practices vary widely—even within the same organization collecting and storing data. Metadata refers to structured information that describes, explains, or contextualizes a dataset to enable discovery and understanding of what the dataset contains. Participants consistently expressed difficulty locating datasets due to inconsistent metadata, minimal semantic tagging, and the absence of shared data catalogs. Attendees also noted the lack of AI-driven tools or automation in metadata creation, contributing to manual bottlenecks, resulting in diminished discoverability.

As part of the pre-summit data collection, organizations submitted their data dictionaries. Analysis revealed that while most dictionaries included basic descriptors such as data type, format, and source, few went beyond these minimal fields. More advanced metadata elements, such as references to ontologies, data provenance, and relationships between datasets, were rarely present, highlighting a clear gap in metadata maturity.

While most organizations at the summit had begun within organizational internal efforts to improve metadata quality, few had consistent policies guiding metadata creation across organizations, and even fewer had externally shareable catalogs. These insights painted a picture of high variability and a strong desire for collaborative approaches.

**Opportunities and Key Recommendations:** Participants identified the following opportunities to strengthen dataset findability through technical, policy, and cultural interventions:

#### Technical opportunities to enhance dataset findability

- **Utilize AI technologies for automated metadata extraction** and unified catalog aggregation, enabling efficient scraping and harmonization of existing data inventories.
- **Implement AI-driven tools to generate fundamental, automated quality indicators**—such as measures of data completeness and missingness—to provide users with quick assessments of dataset reliability.

As AI continues to evolve rapidly, the funding organizations present saw value in pooling resources to collaboratively invest in pilot projects, rather than committing to a specific technology stack at this stage.

#### Policy opportunities to enhance dataset findability

- **Develop and adopt standardized metadata schemas** incorporating common fields (e.g., task, modality, timepoints) and align these with shared taxonomies and ontologies to facilitate federated discovery and dataset integration across repositories.
- **Establish a federated, self-registration metadata catalog**—modeled on platforms like the European Platform for Neurodegenerative Disease (EPND) or ClinicalTrials.gov—that enables data stewards to expose searchable metadata without relinquishing control of the underlying data, ensuring broad and current participation.
- **Introduce quality assessment mechanisms including:**
  ○Voluntary expert review processes that provide independent certification of dataset quality;
  ○Crowdsourced rating systems allowing users to evaluate datasets on usability, clarity, and other attributes; and
  ○Dashboard displays of dataset DOIs and citation metrics to serve as simple, transparent indicators of dataset value and reuse.

**Figure 2.**
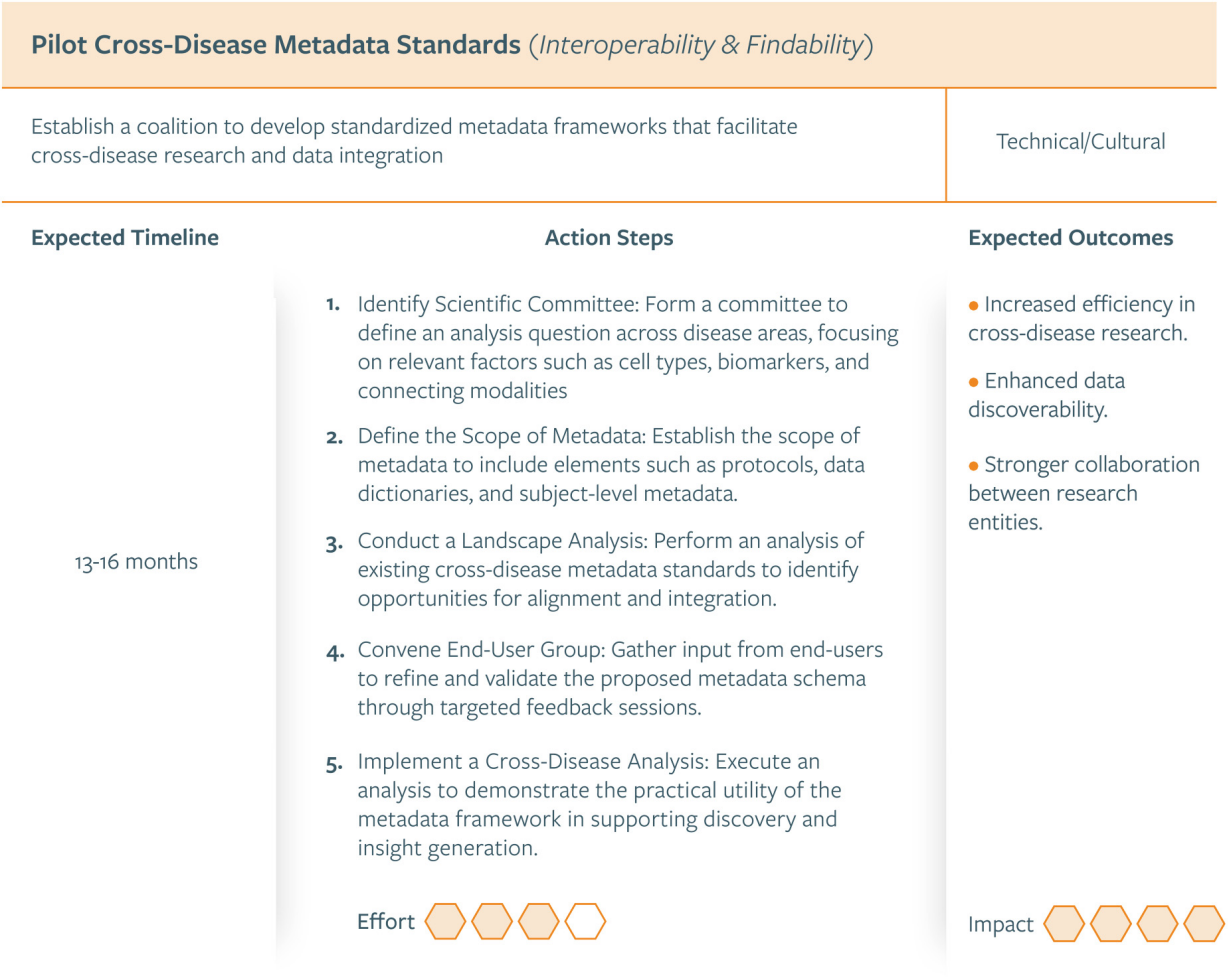
Action plan generated at the summit around piloting cross-disease metadata standards.

Participants prioritized forming a coalition to develop and adopt standardized metadata schemas that enhance cross-disease research data integration and discoverability across organizations. During the summit, participants formed the action plan **(see** Figure 2) for how funders could facilitate metadata standards across diseases to improve findability and interoperability. While initial coordination may be complex, the long-term benefits include greater interoperability and research efficiency. As part of this effort, participants discussed prioritizing the design and execution of a meta-analysis focused on a shared scientific question, using relevant data housed in multiple organizations’ repositories as a practical use case to guide the development of a common metadata schema across datasets. Grounding the work in a real research question was seen as a way to show immediate value and accelerate iterative improvements to the shared schema.

#### Cultural opportunities to enhance dataset findability

- **Increase awareness and adoption of centralized data catalogs** through proactive outreach efforts such as presentations at conferences, targeted communications, and direct engagement with stakeholder communities.
- **Recognize that visibility and trust often outweigh technical features i**n driving dataset usage, highlighting the importance of sustained promotion and community-building activities.

Participants also saw an opportunity to continue to engage in summits such as this one to help cross-promote each other's dataset collections listed in Table 3.

### Accessibility: providing clear, reliable access to data and associated documentation, in accordance with legal, ethical, and security considerations

**Shared Challenges:** Accessibility emerged as a particularly complex dimension, shaped by a myriad of legal, technical, and cultural challenges. During pre-summit interviews, participants reported a patchwork of policies and processes across their institutions for managing access to datasets—particularly those containing sensitive human health data. These processes were often constrained by international regulations such as the European Union's General Data Protection Regulation (GDPR),^48^ variable internal review timelines, and inconsistent documentation practices.

A focal point of the pre-summit research was the review and comparison of Data Use Agreements (DUAs). While most organizations used DUAs to govern data access, there was little uniformity in format, content, or implementation. Some DUAs were highly legalistic and inflexible, while others were informal or adapted on a case-by-case basis. Participants identified that such inconsistency hindered collaborative research efforts, often requiring weeks or months of negotiation to resolve access requests. Interviewees also expressed frustration over the lack of centralized DUA tracking, which created redundant processes and internal inefficiencies.These shared challenges highlight a need for shared tools and governance frameworks that accommodate institutional diversity while improving efficiency.

**Opportunities and Key Recommendations:** Participants identified the following opportunities to strengthen dataset accessibility through technical, policy, and cultural interventions:

#### Technical opportunities to enhance dataset accessibility

- **Implement secure, federated data enclaves** that enable analysis within local jurisdictions while maintaining privacy and compliance (e.g., adapting tools like the AD Workbench).
- **Support cloud adoption through cost transparency and incentives**, such as institutional billing accounts, grant-supported engineering costs, and platform partnerships to reduce data egress fees.
- **Develop a ‘researcher passport’ model** to streamline researcher authentication across repositories, reducing repetitive access requests and enabling more rapid collaboration.
- **Support the development of cloud-agnostic access tools** that allow researchers to interact with datasets across different hosting environments without major reconfiguration.

**Figure 3.**
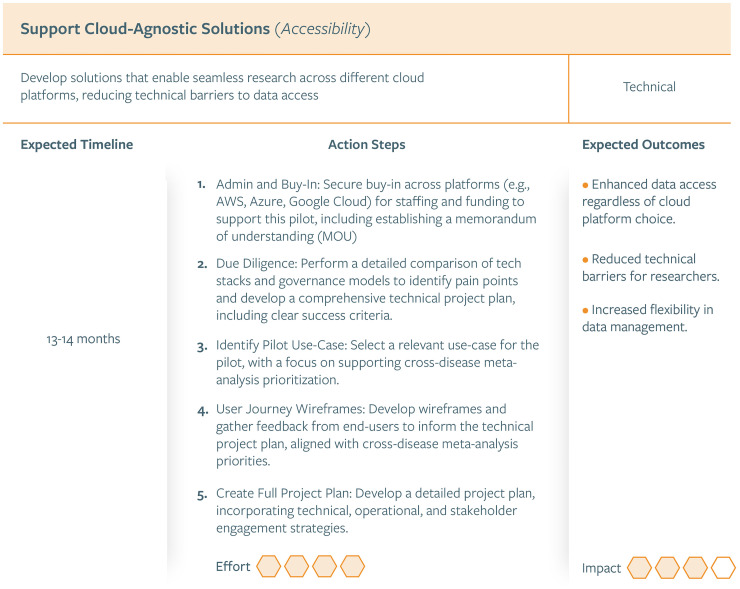
Action plan generated at the summit around developing cloud-agnostic tools.

As funder representatives, there was general alignment around explicitly supporting cloud computing costs in grant budgets to discourage reliance on local-only data analysis environments. Participants also prioritized the development of cloud-agnostic tools enabling analysis across datasets hosted on different platforms (e.g., AWS, Azure, Google Cloud) as a top priority. For an overview of the action plans discussed, see **Figure 3.** Recognizing the potential for a collaborative meta-analysis use case, organizations discussed piloting an effort to bring select datasets into a unified analytical environment, potentially testing a “researcher passport” model within the pilot scope. While considered high-impact, this initiative would require significant time, resources, and leadership buy-in. The next step is for organizational representatives to secure internal support before moving forward.

#### Policy opportunities to enhance dataset accessibility

- **Establish a cross-organization best-practices working group** to share lessons learned and resources across organizations while navigating legal barriers, reducing institutional risk, and clarifying consent requirements.
- **Create shared DUA templates** with standard clauses and adjustable modules tailored to a range of research contexts. These could serve as a starting point for bilateral negotiations and reduce legal overhead.
- **Streamline DUA/ processes** by embedding workflows into platforms, using parameterized agreements with simple checklists, and providing legal clarity directly on data access pages. Alignment with IRBs and clear communication with participants about data use were also emphasized.
- **Adopt open consent frameworks** or pursue **complete anonymization** where feasible to facilitate global data reuse without regulatory delays.

**Figure 4.**
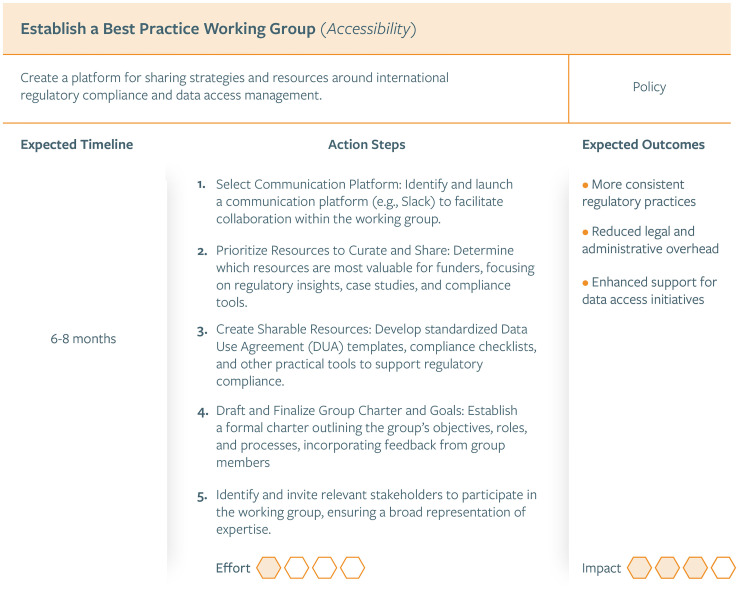
Action plan generated at the summit around establishing a best practice working group.

Of these recommendations, the opportunity prioritized by the group was the formation of a coalition to share tools and practices that reduce duplication of effort and enhance regulatory compliance, data access, and administrative efficiency. See Figure 4 for the action plan around establishing a best practices working group.

#### Cultural opportunities to enhance dataset accessibility

- **Build trust and reduce perceptions of data imperialism** through shared authorship, support for low- and middle- income country investigators, and transparency around data use and reuse.
- **Educate users with onboarding packages and toolkits** to increase user acceptance and demand and lower barriers through communication and training.

Funders discussed requiring grantees conducting meta-analyses to upload analysis notebooks to the data platform as a funding condition. This would help improve usability by providing future users with documented examples that could assist with onboarding and adopting the platform.

### Interoperability: facilitating the integration and exchange of information across diverse platforms through the use of shared standards and formats

**Shared Challenges:** Interoperability—arguably the most technically complex pillar of the FAIR framework—was consistently described during pre-summit interviews as both the greatest challenge and the area with the most untapped opportunity. Interoperability in practice includes both *data harmonization* (“reconciling…data in formats that are compatible and comparable”)^49^ and *platform interoperability* (“open standards are a necessary but not sufficient condition to achieve health data interoperability. The ecosystem of open-source software needs to be considered …”).^50^

Pre-summit discussions revealed that despite strong individual investments in data infrastructure, cross-disease and cross-platform interoperability remains difficult due to inconsistent metadata standards, fragmented infrastructures, and differing technical architectures (programming environments, data storage formats, cloud platforms). Integration is further complicated by modality-specific inconsistencies (e.g., neuroimaging vs. biospecimen vs. wearable data) and limited engineering capacity. In-person discussions centered around the absence of a shared baseline for how to build or evaluate interoperability. Participants also raised concerns about duplicated efforts in developing in-house tools and the absence of centralized knowledge-sharing mechanisms. Few organizations reported dedicated budget lines or long-term strategic plans targeting data integration or schema alignment.

**Opportunities and Key Recommendations:** Participants identified the following opportunities to strengthen dataset interoperability through technical, policy, and cultural interventions:

#### Technical opportunities to enhance dataset interoperability

- **Promote metadata-level harmonization** through shared schema documentation, alignment around core fields (task, modality, timepoint), and ontology mapping.
- **Invest in cloud-agnostic platforms and middleware t**hat connect siloed datasets and simplify data portability. Brokerage or concierge-style services were suggested to guide users through available data.
- **Highlight maturity modeling** to track where modalities or platforms stand on the path to interoperability. This can help prioritize investment—distinguishing between areas that need standards development versus those needing broader adoption.
- **Share protocols, pipelines, and schemas publicly,** encouraging reuse, transparency, and eventual convergence toward widely accepted standards.

Similar to the accessibility recommendation discussions, there was an emphasis on having funders require comprehensive technical documentation from awardees as a condition of funding for data collection and analysis efforts, and support for facilitating design grants that could support work around metadata level harmonization before collection efforts begin.

#### Policy opportunities to enhance dataset interoperability

- **Pilot cross-disease coalitions to define metadata standards** and explore interoperability via real-world use cases. Parkinson's, Alzheimer's, and related neurodegenerative diseases were seen as an ideal starting point, where a real-world analysis project could be focused on a common cross-disease modalitiy like imaging or omics, enabling practical testing and refinement.
- **Embed interoperability requirements into funding mechanisms,** such as mandating the use of community-approved repositories and requiring interoperability planning in experimental design.
- **Professionalize data integration support,** shifting some engineering work out of academia and into funded service roles, such as solutions engineers, data managers, and technical writers supported by consortia.
- **Incorporate dedicated budget lines for interoperability** in grant proposals to support long-term integration work and reduce reliance on ad hoc efforts.

Echoing the discussions on findability, participants emphasized the importance of supporting a coalition to promote metadata-level harmonization, beginning with a pilot use case for cross-disease analysis. Funders also discussed requiring that data collection and analysis grants include dedicated data managers as a budgeted personnel line item, ensuring each team has experts embedded within the team to champion dataset curation and maintain deep familiarity with the relevant platforms.

#### Cultural opportunities to enhance dataset interoperability

- **Normalize a “good enough” standardization mindset** to accelerate adoption, citing examples like Brain Imaging Data Structure (BIDS) where imperfect but pragmatic models have gained wide traction.^^51^^
- **Establish communities of practice** or working groups to maintain momentum, encourage shared learning, and promote reuse of proven interoperability approaches.

The interoperability discussions made clear that while no single solution could address the breadth of technical diversity, supporting a collective movement toward modular standards, strategic resourcing, and shared community ownership offers a viable and compelling path forward. Participants expressed strong interest in establishing a community of practice on interoperability, bringing together technical staff and data managers from participating research organizations to share lessons learned and promote the adoption of common frameworks across the broader research community.

### Reusability: supporting data reuse by ensuring clarity, completeness, and consistency in both the data and its associated metadata

**Shared Challenges:** Reusability was viewed as both an aspirational goal and a cultural challenge. During pre-summit interviews, many participants shared that their organizations struggled to track or measure data reuse effectively. While data was often made available for others to access, there was little infrastructure in place to monitor whether or how it was being reused—let alone to assess the impact of that reuse.

Interviews also highlighted cultural barriers to reuse, such as the reluctance to share scripts, code, and protocols in reproducible formats. Concerns about misinterpretation, lack of attribution, or additional support burdens often discouraged researchers from providing these supplemental materials. While model programs for technical skillbuilding and support that enhance data reusability were identified (e.g., The Carpentries,^52^ McGill's Digital Research Services,^53^ GP2's Learning Platform^54^), attendees noted that individual PDRD researchers may not have the time or interest in developing this secondary set of expertise atop their existing disciplinary specialty. At the same time, there was strong alignment among participants that improving data reuse is essential to achieving the full value of public and private investments in research data.

The summit further explored these challenges, beginning with a participant alignment activity around the level of reuse tracking and incentive structures currently in place within their organizations. Responses revealed that most groups had informal mechanisms—such as anecdotal reporting or post-publication tracking—but few had systematic ways of evaluating reuse or encouraging contributors to document their work in reusable formats.

**Opportunities and Key Recommendations:** Participants identified the following opportunities to strengthen dataset reusability through technical, policy, and cultural interventions:

#### Technical opportunities to enhance dataset reusability

- **Develop automated tools to track reuse**, including DOI citations, inclusion in meta-analyses, and shared analytical outputs. This could include AI-driven landscape mapping of how datasets are used across publications and platforms.
- **Create usability metrics and taxonomies** to classify datasets (e.g., by domain, preclinical/clinical status, model used), improving discoverability and contextual understanding.
- **Incorporate dataset provenance and FAIRness assessments** as part of submission and cataloging, including scorecards and “stamps” for documentation quality, ease of use, and adherence to standards.
- **Invest in infrastructure to support code and model sharing**, with platforms like GitHub, Code Ocean, and HuggingFace backed by funder-driven training, doc-a-thons, and community rating systems.
- **Foster engagement through user-centered design**, including biology-friendly UIs, onboarding materials, code notebooks, and data challenges to draw new users in.

Opportunities surfaced through discussions of successful community-led initiatives that reward transparency and reproducibility. Not all opportunities can be addressed by funders alone, but near-term actions—such as hosting data challenges to attract new users and co-funding infrastructure to support code and model sharing—were seen as feasible first steps.

#### Policy opportunities to enhance dataset reusability

- **Develop common metrics and dashboards** to assess data reuse, including citations, software forks, downloads, and integration into new analyses.
- **Incentivize better citation practices** by requiring templated data citations in publications and pushing journals to adopt open access and platform citation policies.
- **Create recognition and reward systems** that celebrate meaningful contributions to reuse, including badges, co-authorship, and grant incentives.
- **Promote the development of an Sharing-index**—a reuse-specific metric that complements the traditional H-index, to analyze how often datasets have been re-used.
- **Fund re-analysis and meta-analysis projects**, independent of initial data generation, to spur new insights and demonstrate value through targeted data challenges.

As John Naisbitt famously said, “We are drowning in data but starving for knowledge.” As highlighted in the technical recommendations, research organizations prioritizing re-analysis and meta-analysis of existing datasets is a critical step toward transforming abundant data into meaningful insights.

#### Cultural opportunities to enhance dataset reusability

- **Encourage community feedback models** (e.g., “works for me” ratings, thumbs-up systems), enabling lightweight validation of dataset utility and surfacing valuable resources.
- **Build coalitions to share best practices**, form working groups, and highlight success stories that reinforce data reuse as a core research output.

Consistent with findings from librarianship, reusability discussions illustrated that while the technical tools for sharing PDRD data already exist, cultural and organizational supports remain underdeveloped and are critical to successful community activation in service of data reusability.^55^ Building upon the emerging theme from all the FAIR principles, a best practices working group that would help with building coalitions not just within the organizations present at the summit, but the broader research community at large, was seen as a high-priority, high-impact next step.

## Conclusions

The outcomes of the PDRD Data Interoperability Summit 2025 reveal both the complexity of implementing FAIR data principles in the neurodegenerative disease research space and the eagerness of the private funder community to address these challenges collaboratively. Participants were glad to start the conversation with each other but also interested in expanding the organizational representation to include other research organizations that may have a large footprint in the neurodegenerative space. Across all four pillars of FAIR, there was an unmistakable sense of convergence on the core barriers. Rather than treating these issues independently, participants emphasized the value of holistic, cross-cutting interventions – those that blend technical development with policy reforms and cultural incentives. This system-level lens was a key theme throughout the summit and became a guiding orientation for many of the following recommendations.

One of the summit's most notable outcomes was the sheer breadth of recommendations generated across technical, policy, and cultural dimensions. This diversity reflects not only the scale of need but also the appetite for innovation within the community. Participants brought not only strategic ideas, but also practical, context-specific proposals rooted in lived experience with data governance, tool development, and research workflows.

While initial action plans were developed during the summit to catalyze immediate progress, the remaining recommendations present a roadmap for long-term transformation. These priorities were not selected based on theoretical appeal alone, but through a participant-led scoring and sorting process using an impact-versus-effort framework. Critically, this summit affirmed that change is not only possible, but already underway. Multiple participants shared existing efforts and pilot projects that align with FAIR principles—evidence that the ecosystem does not need to start from scratch, but instead must better connect, coordinate, and amplify what already works. Projects supporting federated search, modular metadata schemas, or cross-institutional analytics already exist, but lack the scaffolding of shared strategy or infrastructure investment. In many cases, the missing ingredient is not technology, but shared governance, standardization, and strategic funding mechanisms.

A key takeaway from the summit was the importance of avoiding “boiling the ocean.” Participants consistently emphasized the need for phased implementation strategies, grounded in specific pilot use cases and measurable outcomes. The risk of over-engineering or aiming for universal solutions too early was flagged as a common cause of stalled initiatives. Instead, the group championed pilots, modular frameworks, and feedback loops—prioritizing progress over perfection. This mindset was reflected in how the final action plans were scoped: each with clear boundaries, testable hypotheses, and pathways for evaluation.

In sum, the 2025 PDRD Interoperability Summit surfaced a clear message: the scientific community has the will and capacity to transform how neurodegenerative disease research data is shared and reused. But doing so will require more than tools and templates—it will require a coalition of aligned actors working with intention, humility, and mutual accountability. The recommendations and action plans developed here are a strong step forward, but only the beginning of a longer journey toward a truly FAIR research ecosystem—one that not only advances discovery but also reflects the shared values of transparency, collaboration, and equity. The summit reinforced that FAIR is not merely a set of technical guidelines. It is a framework for values-driven collaboration—grounded in equity, openness, and shared stewardship of data as a public good. As such, FAIR implementation must be inclusive, iterative, and attentive to both the technical and human dimensions of data sharing.

As research funders, we can support FAIR data sharing through coordinated action across technical, policy, and cultural domains. On the technical front, we can invest in shared infrastructure, adopt standard tools and formats, and embed interoperability as a strategic priority within our organizational roadmaps. From a policy perspective, we can collaborate with fellow funders to co-develop clear, aligned data policies and governance frameworks that support consistent and responsible data sharing. Culturally, we can incentivize good practices by recognizing contributions to data sharing and by fostering transparency and collaboration across our shared initiatives. The positioning of this workshop as a summit between private funders of research into neurodegenerative diseases provides a path to enacting systemic change and improving data interoperability. Agreement between funders on strategic directions and data governance requirements, prioritization of budget line items that support data interoperability and not just data generation and analysis, and shared understanding of best practices that recipients can and should utilize, will help create a framework for interoperability to amplify the scientific and translational impacts of their research investment. Moving forward, the momentum generated by the summit must be sustained. This will require not only follow-through on the action plans, but also a governance model that supports accountability, adaptation, and expansion. It will require new mechanisms for aligning funding priorities, sharing learnings, and measuring progress. And it will require a continued commitment to convening—not just once, but regularly—as a mechanism for collective reflection, recalibration and accountability to make change happen.

## Supplemental Material

sj-jpg-1-pkn-10.1177_1877718X261430266 - Supplemental material for Insights and priorities from the 2025 private funders’ Parkinson's disease and related disorders large footprint data interoperability summit Supplemental material, sj-jpg-1-pkn-10.1177_1877718X261430266 for Insights and priorities from the 2025 private funders’ Parkinson's disease and related disorders large footprint data interoperability summit by Leslie C Kirsch, Emily Baxi, Mukta Phatak, Francis Jeanson, Dave Alonso, Cornelis Blauwendraat, Matthew CH Boersma, Patrick Brannelly, Matthew HS Clement, Amy Easton, Hilary Jenkins, Ritu Kapur, Tyler Mollenkopf, Bryce Pickard, Amy Rommel, Carol L Thompson, Jonathan White and Sonya B Dumanis in Journal of Parkinson's Disease

sj-xlsx-2-pkn-10.1177_1877718X261430266 - Supplemental material for Insights and priorities from the 2025 private funders’ Parkinson's disease and related disorders large footprint data interoperability summit Supplemental material, sj-xlsx-2-pkn-10.1177_1877718X261430266 for Insights and priorities from the 2025 private funders’ Parkinson's disease and related disorders large footprint data interoperability summit by Leslie C Kirsch, Emily Baxi, Mukta Phatak, Francis Jeanson, Dave Alonso, Cornelis Blauwendraat, Matthew CH Boersma, Patrick Brannelly, Matthew HS Clement, Amy Easton, Hilary Jenkins, Ritu Kapur, Tyler Mollenkopf, Bryce Pickard, Amy Rommel, Carol L Thompson, Jonathan White and Sonya B Dumanis in Journal of Parkinson's Disease
